# An investigation into the comfort and neural response of textured visual stimuli in pediatric SSVEP-based BCI

**DOI:** 10.1038/s41598-025-09540-8

**Published:** 2025-07-18

**Authors:** Emily Schrag, Daniel Comaduran Marquez, Adam Kirton, Eli Kinney-Lang

**Affiliations:** 1 Department of Pediatrics, Cumming School of Medicine, University of Calgary, Calgary, AB Canada; 2 Department of Neuroscience, University of Calgary, Calgary, AB Canada; 3 Calgary Pediatric Stroke Program, Alberta Children’s Hospital, 28 Oki Drive NW, Calgary, AB Canada; 4 Alberta Children’s Hospital Research Institute, Calgary, AB Canada; 5 Hotchkiss Brain Institute, University of Calgary, Calgary, AB Canada; 6 Department of Clinical Neurosciences, University of Calgary, Calgary, AB Canada; 7 Department of Biomedical Engineering, University of Calgary, Calgary, AB Canada

**Keywords:** Brain–computer interface, BCI, SSVEP, Comfort, Pediatric BCI, Paediatric research, Outcomes research

## Abstract

Steady-state visual evoked potential (SSVEP)-based brain–computer interfaces (BCIs) are widely used due to their reliability and possible training-free setup. Common SSVEP stimuli are high contrast and solidly colored, potentially causing discomfort and visual fatigue, particularly when high stimulation frequencies are employed. To address this, textured stimuli, which may evoke visual responses in higher processing systems, have been proposed as an alternative to conventional flashing stimuli. We evaluate the effectiveness of textured stimuli for SSVEP-based BCIs by examining both user comfort and neural responses across different EEG channel subsets. Neurotypical participants aged 5–18 (*n* = 35, 57% female) were exposed to traditional and textured stimuli at three frequencies (9, 14, and 33 Hz) and asked to report perceived comfort. While textured stimuli were consistently rated as more comfortable, especially at lower frequencies, signal-to-noise ratio analysis indicated that they did not enhance neural responses compared to conventional stimuli. Classification accuracy was driven primarily by stimulation frequency rather than stimulus type and there was a sharp decline in accuracy at 33 Hz. These findings suggest that while textured stimuli improve user comfort, their utility in enhancing BCI performance remains unclear, warranting further investigation into stimulus design for SSVEP-based BCIs.

## Introduction

A brain–computer interface (BCI) translates electrical signals recorded from the brain into external actions. BCI systems enable individuals with severe motor impairments to interact with their environment in previously inaccessible ways, such as controlling a computer cursor^1^. Electroencephalography (EEG) is a common, non-invasive method of recording electrical activity from the brain using electrodes on the scalp. The steady-state visual evoked potential (SSVEP) is one commonly used method of BCI control^2^. The SSVEP is elicited in the brain when an individual observes a stimulus, typically on a computer screen, flashing at a specific, constant frequency^2^. The frequency of the flashing stimulus synchronizes with the observer’s EEG signal, which can be processed and used to identify which stimulus object the participant was focusing on.

High contrast, solid color stimuli reliably modulate activity in the primary visual cortex and are the typical choice for eliciting strong SSVEP signals for use in a BCI system^3^. While these stimuli produce robust SSVEP signals, they also contribute to visual fatigue and physical discomforts^4^ such as drowsiness^5^ and headaches^6^. Visual fatigue from high contrast flashing stimuli can lead to a decline in attention, diminishing the SSVEP signal and subsequently reducing BCI performance^5^.

The use of BCI technology in children is a highly underdeveloped area of research^7^. Millions of young people with severe motor impairments often lack the opportunity to interact with their environment in the same way as their peers. Developing BCI technology with children in mind is crucial to provide such opportunities^8^. The SSVEP is a robust, often training-free BCI control paradigm that has only been explored minimally in a pediatric population. A low rate of task completion has been reported in younger children using a speller SSVEP BCI system at low stimulation frequencies (i.e. close to the alpha band)^9^, highlighting the possible influence of developmental differences in SSVEP performance between children and adults. ^10^ explored pediatric performance in a game-based SSVEP system with promising results. The current experiment serves as an examination for the further development of comfortable SSVEP stimuli which could promote greater adoption of BCI use by children with severe motor impairments.

**Fig. 1:**
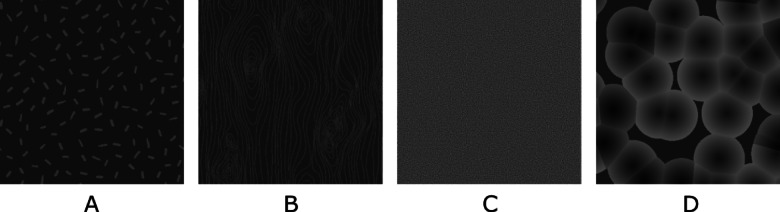
Textures presented as SSVEP flashing stimuli. (**A**) Worms, (**B**) Wood Grain, (**C**) Static, (**D**) Voronoi.

The neural mechanisms underlying the visual processing of complex textures remain largely unknown. Complex textures contain a large volume of information, making it difficult to synthesize experiments that can comprehensively evaluate multiple aspects of a textured image simultaneously^11^. Despite this complexity, several aspects of the visual processing of textures have been explored. ^12^ demonstrated that neurons in areas V1 and V2 of the visual system exhibit differential responses to artificial and naturalistic images. Specifically, the response of V2 to artificially produced stimuli (e.g., non-natural images) is similar to the response of V1^12^. When presented with patterns derived from natural images (e.g., honeycomb, beans, etc.), however, V2 neurons show a selective response^12^. The proposed textured stimuli are representative of natural images (Fig. 1) so it is expected that there will be an increased response in area V2. Another study investigated the difference in neural response to random and correlated textured images. Random textured images displayed black and white pixels organized randomly, while correlated textured images displayed organized groups of black and white pixels arranged into extended contours and blocks of pixels of the same color^13^. Presentation of correlated textured images elicited an increase in activity in the anterior fusiform, lingual, and middle temporal gyri that was not observed in response to random textured images^13^. Applying the same type of classification to the textured stimuli used in this study (Fig. 1), the Static stimulus is categorized as a random textured image, while the Worms, Wood Grain, and Voronoi stimuli are categorized as correlated textured images.

It remains to be determined if low contrast, textured stimuli can produce SSVEP signals strong enough to control a BCI system. A unique response measured by electrodes over the occipital cortex is expected, arising from area V2, in contrast to the characteristic response of area V1 to solid color, high contrast stimuli. Based on their classification as correlated textured images, it is expected that stimulation by the Worms, Wood Grain, and Voronoi stimuli will activate the higher-level visual processing mechanisms that will be measured by electrodes over the temporal lobes.

We evaluated a novel set of low contrast textured stimuli presented at three different frequencies to determine the comfort and efficacy of textured stimuli compared with solid color high contrast stimuli. We assessed whether the proposed textured stimuli were perceived as more comfortable than state-of-the-art high contrast stimuli and whether they produce sufficient SSVEP response to control a BCI system. We anticipated that the textured stimuli would be perceived as more comfortable due to their low contrast nature. Further, we hypothesized that the loss in SSVEP response in the primary visual cortex (V1) due to the lower contrast content of the textured stimuli would be compensated for by additional responses in higher cortical areas, specifically areas V2 and the temporal lobe. This in turn would result in an adequately strong SSVEP response capable of being used in a BCI-like environment. We additionally propose the use of such textured stimuli in future SSVEP-based BCI systems to alleviate discomfort and fatigue.

## Methods

### Participants

Thirty-five participants aged 5–18 years (12.3 +/- 4.2 mean +/- SD, 57% female) who did not take prescription medication and had no history of neuropsychiatric disorders or neurodevelopmental conditions were recruited from the Healthy Infants and Children’s Clinical Research Program (HICCUP), a community-based healthy controls recruitment program for research^14^. Informed assent and parental consent were obtained from all participants. This study was performed in accordance with the University of Calgary’s Conjoint Health Research Ethics Board’s policies. This study was approved by the University of Calgary’s Conjoint Health Research Ethics Board under ID: REB23-1232.

### Visual stimulus characteristics

#### Frequency

Three frequency values were selected, representing the entire frequency range of typical SSVEP stimuli: 9.$\:\overline{09}$, 14.28 and 33.$\:\overline{3}\:$Hz, hereafter referred to as 9, 14, and 33 Hz. Selected frequency values represent low (< 12 Hz), medium (12–30 Hz), and high (> 30 Hz) SSVEP frequency ranges^15^.

#### Contrast, color & pattern reversal

Neutral solid color stimuli were presented at the minimum and maximum contrast ratios against a solid black background. The minimum contrast stimulus presents a dark grey box against a black background (2:1) and was chosen for its perceived comfort^16^. The maximum contrast stimulus, presented as a white box against a black background (21:1), has been shown to reliably induce SSVEPs for BCI systems^17^ and is typical of SSVEP stimuli reported in BCI literature^18–20^. A checkerboard reversal pattern stimuli was included due to its established response characteristics when used in SSVEP systems^21^.

#### Textures

Four unique textured stimuli were additionally created using the Unity3D Technologies Game Engine^22^: a simple random pattern with small components (Worms, Fig. 1A), similar to patterns used in commercial BCI systems^23^; a natural texture (Wood Grain, Fig. 1B); a visual representation of static noise (Static, Fig. 1C); and a pattern with conjoined boundaries based on a recurring natural phenomenon (Voronoi, Fig. 1D).

### Stimulus presentation

#### Stimuli

In total, seven types of stimuli were presented: solid color stimuli at maximum (21:1) and minimum (2:1) contrast levels, a checkerboard pattern, and four texture conditions (Worms, Wood Grain, Static, and Voronoi). The checkerboard stimulus was presented as a pattern reversal, while all other stimuli were presented as flashing.

#### Stimulus presentation protocol

Stimuli were presented using the Unity3D software^22^, and an extension of the BCI-Essentials Unity open-source package^24^. All stimuli were presented on a 32” LG monitor with a refresh rate of 100 Hz.

**Fig. 2:**
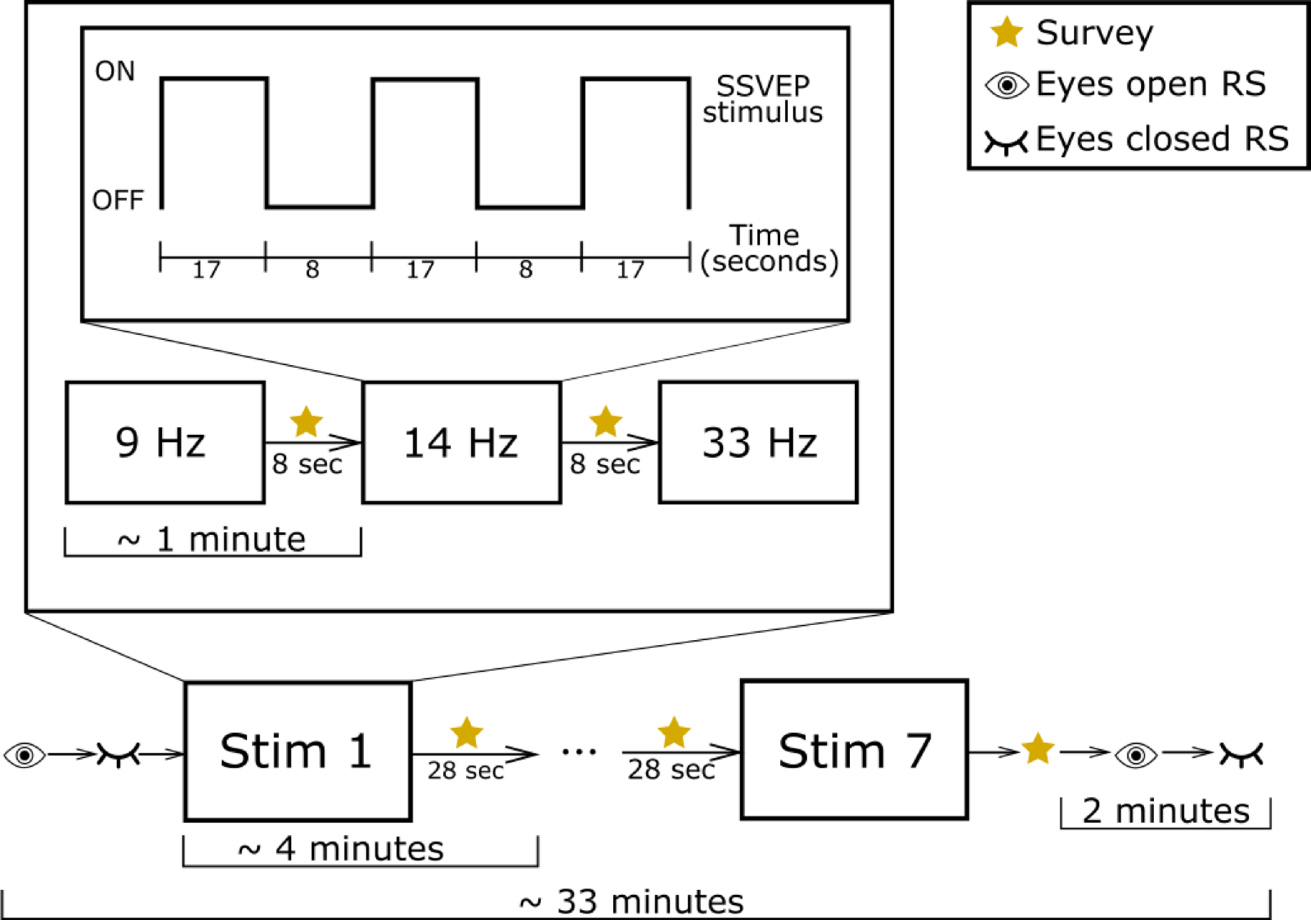
Stimulus presentation protocol. Resting state EEG data recording is abbreviated as “RS”.

Timing of the stimulus presentation is illustrated in Fig. 2. Following a verbal cue from the researcher, participants opened their eyes and observed the first flashing stimulus. The stimulus flashed against the solid black background for 17 s, followed by a period of 8 s where only the background was visible. Flashing resumed after another 8 s, and the pattern continued until 1 min and 7 s had elapsed (three 17 s “on” periods recorded for each stimulus). There was an 8 s rest period where the participant was prompted verbally and by text on the screen to report the comfort of the stimulus from a 7-point Likert scale ranging from 1 (most comfortable) to 7 (least comfortable). The stimulus began to flash at the next frequency value following the 8 s break and this pattern repeated until the stimulus was presented at all three frequencies. Following the completion of a set, there was a 28 s rest period which was also used to report comfort of the last stimulus in the frequency set. At the beginning and end of the stimulus presentation session, one minute of eyes open resting state and eyes closed resting state EEG data was recorded.

During stimulus presentation, participants were seated in a laboratory setting with overhead fluorescent lights on. Being located in a hospital, the indoor temperature and humidity were controlled between participants. The same location was used for all participants, helping minimize potential environmental variations. The total time for the stimulus presentation session was around 33 min. There were setup and cleanup times of approximately 10 min each. The entire session lasted between 50 and 60 min for each participant.

### EEG data acquisition

EEG data was recorded with a sampling rate of 256 Hz using the gel-based g.tec g.GAMMAsys system, g.USBamp amplifier, and g.GAMMAcap headcap. A montage of 16 electrodes (Oz, POz, Pz, Cz, O1, O2, PO7, PO8, P3, P4, P7, P8, T7, T8, C3, C4) was placed in accordance with standard 10–10 system locations, with a common reference electrode at Fpz.

### EEG data processing

Data was analyzed based on established best practices for EEG signal processing (analysis code available at easy-on-the-eyes^25^). First, the EEG data underwent visual inspection to remove EEG channels with significant noise levels. The data was bandpass filtered (0.5–35 Hz), followed by an independent component analysis (ICA) that identified and eliminate spurious features contributing to noise in the signal^26^. To determine the frequency content of the EEG data, the broadband power spectral density (PSD) was computed and used to calculate the SNR for each desired frequency peak present in the power spectrum of the EEG data based on^27^. The SNR calculation included two harmonics above and below each tested frequency. An SNR value in decibels (dB) was calculated for each unique stimulus for each participant.

Four EEG channel subsets were identified, based on typical brain anatomy. The “All” channel subset included all 16 channels in the electrode array used in the study; the “O” channel subset included channels O1, Oz, and O2; the “T” channel subset included channels T7 and T8; and the “OT” channel subset included the union of the “O” and “T” subsets, i.e. O1, Oz, O2, T7, and T8.

To determine the practical performance of the stimuli in an offline BCI-like system, a series of four state-of-the-art SSVEP classifiers were investigated using the stimulus “on”-block of data and the “O” channel subset (i.e. O1, Oz, and O2 electrodes only). The classifiers investigated, from the SSVEP Toolbox^28^ were:

1. Filter-bank canonical correlation analysis (fbCCA)^29^.
2. Minimum energy combination (MEC)^30^.
3. Multivariate synchronization index (MSI)^31^.
4. Riemannian geometry adapted logistic regression (RG-logreg)^32^.

All classifiers were run on bandpass filtered EEG data using “on” block data for each stimulus for calculation of classification accuracy and further divided into “on” block data for each stimulus at each frequency for calculation of sensitivity. The “on” block data for each stimulus consisted of a total of 9 17 s periods.

To assess the classification accuracy of the 4 multi-class classifiers, their performance was assessed across the set of 9 distinct “on” periods for each textured stimulus. Each classifier was tasked with determining the correct frequency class for each “on” period, where the possible classes corresponded to the three stimulation frequencies (e.g., 9, 14, and 33 Hz). For the RG-logreg classifier, a 3-fold cross validation was used to derive a mean CA for each textured stimulus. All other classifiers used no training data and therefore cross-validation was not required. For each textured stimulus, CA was determined by counting the number of correctly classified “on” periods out of the 9 total “on” periods. The reported CA for each stimulus type represents the mean value across all participants.

To determine differences in classifier sensitivity, their performance was assessed by calculating the true positive rate per stimulation frequency for each textured stimulus. The ‘target’ class represented the frequency of interest (ex. 9 Hz) while the ‘non-target’ class represented the other 2 stimulation frequencies (ex. 14 and 33 Hz). For the RG-logreg classifier, a 3-fold cross validation was used to derive a mean sensitivity for each textured stimuli at the different frequencies. All other classifiers used no training data and therefore cross-validation was not required. The sensitivity for each textured stimuli at each frequency is the number of correctly classified “on” periods divided by the total number of “on” periods for each stimulus at each frequency. Since there are only 3 “on” periods per stimulus, the sensitivity has a value of 0, 33, 67, or 100%. The reported sensitivities are the mean of these values across all participants.

### Statistical analysis

SPSS software^33^ was used for statistical analyses. Distribution normality was tested using the Shapiro-Wilk test. SNR and comfort scores were compiled for each stimulus group: maximum contrast (black/white), minimum contrast (black/grey), Worms, Wood Grain, Static, Voronoi, and checkerboard. An additional general texture group was calculated as the average scores for the four individual textured stimuli to compare textures as a group to traditional stimulus types while reducing multiple statistical comparisons.

#### Experiment 1: comfort of textured stimuli

To determine if the textured stimuli were perceived as more comfortable than high contrast, solid color stimuli, we compared comfort scores of minimum and maximum contrast, checkerboard, and average of the four textured stimuli (Experiment 1A). We also compared comfort scores among the four textured stimuli (Worms, Wood Grain, Static, and Voronoi stimuli; Experiment 1B). Two-way repeated measures analyses of variance (RM ANOVA) were used with frequency and stimulus type as independent variables allowing for additional examination of their interaction.

#### Experiment 2: SSVEP response to textured stimuli

To determine if the textured stimuli could produce sufficient SSVEP responses for use in a BCI system we compared SNR values of minimum and maximum contrast, checkerboard, and average of the four textured stimuli (Experiment 2A). We also compared SNR values among the four textured stimuli (Experiment 2B) and evaluated performance of the four individual textured stimuli via a set of four classifiers (Experiment 2C). Three-way RM ANOVAs were used, with frequency, stimulus, and channel subset as independent variables.

For all RM ANOVA results, the Bonferroni correction was used for multiple comparisons, and the Greenhouse-Geisser correction was employed in any case of a violation of sphericity.

## Results

### Experiment 1—comfort differences

#### Experiment 1A−comparison of different stimulus types

Although comfort score data was measured using a Likert scale, and residuals displayed a deviation from the normal distribution, RM ANOVAs were used given evidence that these deviations would be minimal in affecting outcomes of the statistical analysis^34^. In addition, ANOVA has been shown to be robust to deviations from normality when the sample size is larger than 10^35^.

**Fig. 3:**
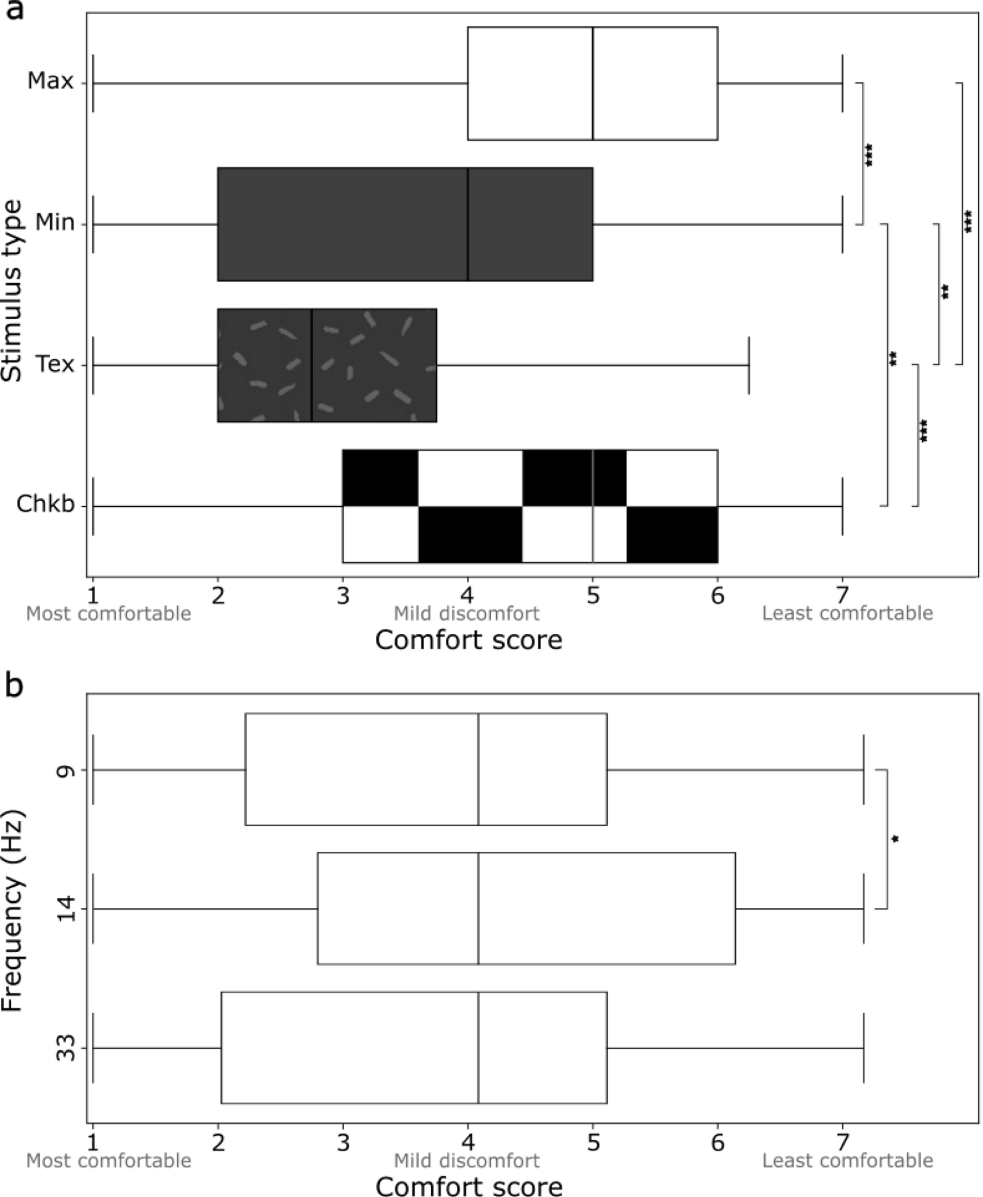
Comfort scores reported from a 7-point Likert scale presented for (**a**) stimulus type and (**b**) frequency (Hz), from most comfortable (1) to least comfortable (7). Boxes represent the interquartile range (IQR) with the vertical line indicating the median comfort score. Whiskers extend to 1.5 times the IQR. Max, maximum contrast; Min, minimum contrast; Tex, texture, and Chkb, checkerboard. Significance after Bonferroni correction indicated by: * for significance *p* <.05; ** for *p* <.01; *** for *p* <.001.

We found main effects of frequency (*F*_(2, 68)_ = 4.16, *p* =.020) and stimulus type (*F*_(3, 102)_ = 34.29, *p* <.001) on comfort score, but no interaction between the two. Pairwise comparison tests revealed that the comfort of textured stimuli was rated as more comfortable than the maximum contrast (*p* <.001), minimum contrast (*p* =.004), and checkerboard stimuli (*p* <.001). Additionally, the minimum contrast stimulus was more comfortable than the maximum contrast (*p* <.001) and checkerboard stimuli (*p* =.001). Stimuli presented at 14 Hz were less comfortable than stimuli presented at 9 H z (*p* =.030). These results are displayed in Fig. 3.

#### Experiment 1B- comparison of individual textured stimuli

**Fig. 4:**
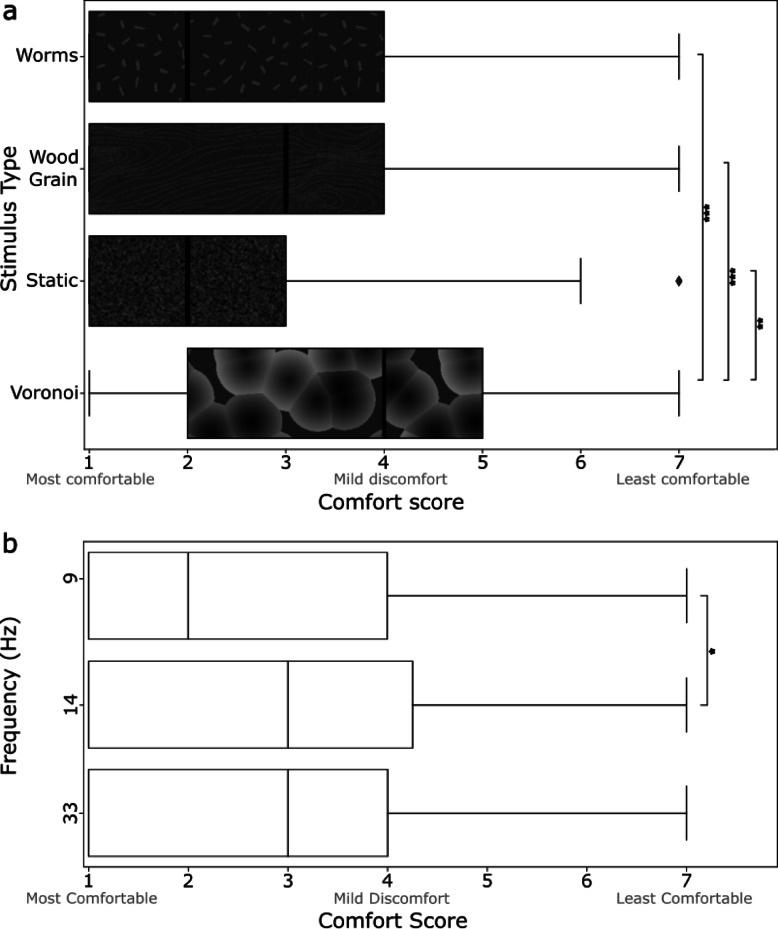
Comfort scores reported from a 7-point Likert scale presented for (**a**) textured stimulus type and (**b**) frequency (Hz), from most comfortable (1) to least comfortable (7). Boxes represent the interquartile range (IQR) with the vertical line indicating the median comfort score. Whiskers extend to 1.5 times the IQR. Significance after Bonferroni correction indicated by: * for significance *p* <.05; ** for *p* <.01; *** for *p* <.001.

We found main effects of frequency (*F*_(2, 68)_ = 3.40, *p* =.039) and stimulus type (*F*_(2.32, 78.93)_ = 9.23, *p* <.001) on comfort score, but no interaction between the two. Mauchly’s test indicated a violation of sphericity for the main effect of stimulus type (X^2^(5) = 0.667, *p* =.021) therefore the Greenhouse-Geisser correction was applied. Pairwise comparison tests showed that the Voronoi stimulus was rated as less comfortable than the Worms (*p* <.001), Wood Grain (*p* <.001), and Static stimuli (*p* =.004). Additionally, stimuli presented at 14 Hz were rated less comfortable than stimuli presented at 9 Hz (*p* =.035). These results are displayed in Fig. 4.

### Experiment 2—SNR differences

#### Experiment 2A—comparison of different stimulus types

A significant three-way interaction between stimulus type, frequency, and channel subset was observed (*F*_(6.37, 216.73)_, *p* <.001). Mauchly’s test indicated a violation of sphericity for this interaction (X^2^(170) = 0.00, *p* <.001), therefore the Greenhouse-Geisser correction was applied.

Investigating the individual components of the three-way interaction, it was found that the interaction of frequency and stimulus type was significant for the O (*F*_(6, 408)_ = 7.86) and OT channel (*F*_(6, 408)_ = 21.56) subsets, but not for the All (*F*_(6, 408)_ = 1.50) and T channel (*F*_(6, 408)_ = 0.22) subsets.

**Fig. 5:**
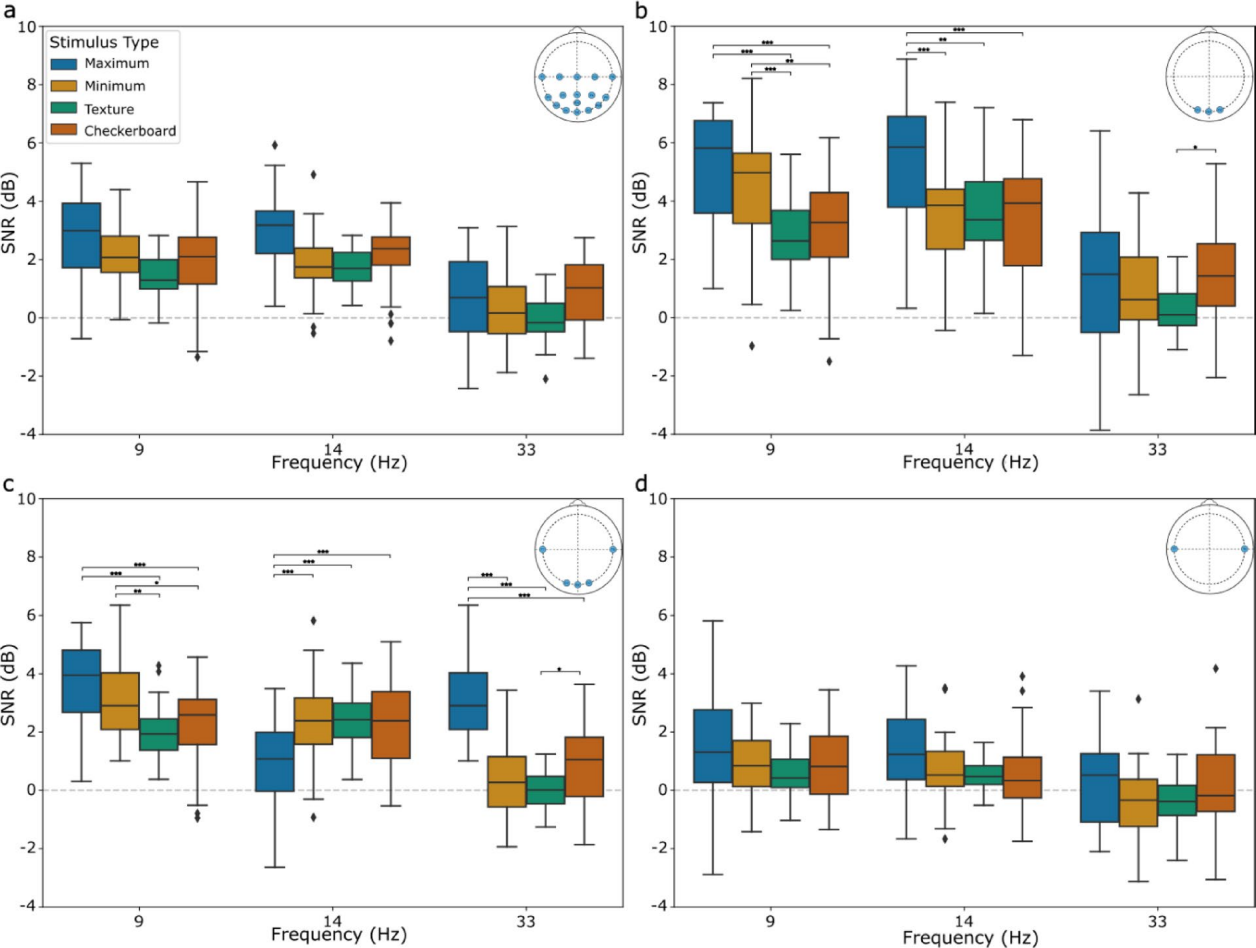
SNR (dB) for each of the 4 visual stimulus conditions at each of the 3 frequencies (Hz) for each channel subset: (**a**) All (**b**) O (**c**) OT (**d**) T. The ‘Texture’ stimulus type indicates the averaged SNR for the 4 textured stimuli and was used to investigate a manageable number of statistical comparisons. Boxes represent the interquartile range (IQR) with the vertical line indicating the median comfort score. Whiskers extend to 1.5 times the IQR. Channel subset locations are provided in the top right of each panel from a top-down view using the standard 10–10 system. Significance following Bonferroni correction is indicated by: * for *p* <.05, ** for *p* <.01, *** for *p* <.001.

For the O channel subset at 9 Hz (*F*_(3, 136)_ = 14.26, *p* <.001), the maximum contrast stimulus had higher SNR values than the checkerboard (*p* <.001) and averaged textured stimuli (*p* <.001). The minimum contrast stimulus also had a higher SNR value than the checkerboard stimulus (*p* =.002) and averaged textured stimuli (*p* <.001). At 14 Hz (*F*_(3, 136)_ = 7.98, *p* <.001), the maximum contrast stimulus had higher SNR values than the minimum contrast (*p* <.001), checkerboard (*p* <.001), and averaged textured stimuli (*p* =.002). At 33 Hz (*F*_(3, 136)_ = 3.51, *p* =.017), the checkerboard stimulus had higher SNR values than the averaged textured stimuli (*p* =.027). Results visualized in the top right panel of Fig. 5.

For the OT channel subset at 9 Hz (*F*_(3, 136)_ = 14.39, *p* <.001), the maximum contrast and minimum contrast stimuli had higher SNR values than the checkerboard (*p* <.001; *p* =.014) and averaged textured stimuli (*p* <.001; *p* =.001). At 14 Hz (*F*_(3, 136)_ = 9.54, *p* <.001), the maximum contrast stimuli had SNR values lower than the checkerboard (*p* <.001), minimum contrast (*p* <.001), and averaged textured stimuli (*p* <.001). At 33 Hz (*F*_(3, 136)_ = 50.91, *p* <.001), the maximum contrast stimuli had SNR values higher than the checkerboard (*p* <.001), minimum contrast (*p* <.001), and averaged textured stimuli (*p* <.001). Additionally, the checkerboard stimulus had SNR values higher than the averaged textured stimuli (*p* =.014). Results visualized in the bottom left panel of Fig. 5.

#### Experiment 2B—comparison of individual textured stimuli

Mauchly’s test indicated a violation of sphericity for the interaction between stimulus type, frequency, and channel subset (X^2^(170) = 0.00, *p* <.001), therefore the Greenhouse-Geisser correction was applied. A significant three-way interaction was observed between stimulus type, frequency, and channel subset (*F*_(6.47, 219.86)_ = 2.74, *p* =.012).

Investigating the individual components of the three-way interaction revealed that the interaction of frequency and stimulus type was only significant for the O channel subset (*F*_(6, 408)_ = 3.47), with All (*F*_(6, 408)_ = 1.09), OT (*F*_(6, 408)_ = 1.14), and T channel (*F*_(6, 408)_ = 0.29) subsets found to be non-significant.

**Fig. 6:**
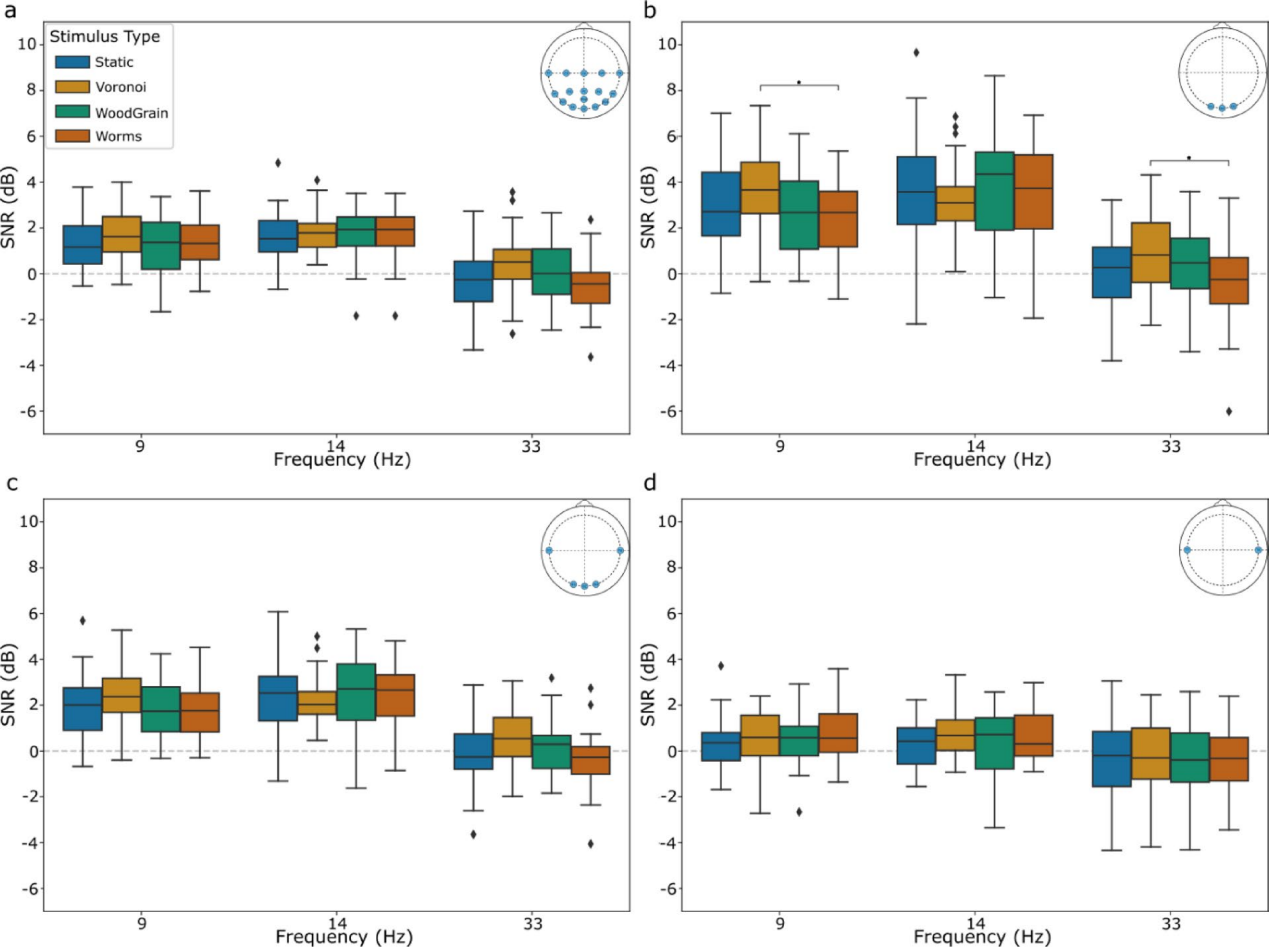
SNR (dB) for each of the 4 visual textured stimulus conditions at each of the 3 frequencies (Hz) for each channel subset: (**a**) All (**b**) O (**c**) OT (**d**) T. Boxes represent the interquartile range (IQR) with the vertical line indicating the median comfort score. Whiskers extend to 1.5 times the IQR. Channel subset locations are provided in the top right of each panel from a top-down view using the standard 10–10 system. Significance following Bonferroni correction is indicated by: * for *p* <.05, ** for *p* <.01, *** for *p* <.001.

For the O channel subset at 9 Hz (*F*_(3, 136)_ = 3.33, *p* =.022), the Voronoi stimulus exhibited a higher SNR value than the Worms stimulus (*p* =.034). At 33 Hz (*F*_(3, 136)_ = 3.62, *p* =.015), the same result was observed (*p* =.011). Results in Fig. 6.

#### Experiment 2C—classification of textured stimuli

To assess the classification accuracy of the 4 multi-class classifiers, their performance was assessed across the set of 9 “on” periods for each textured stimulus. Each classifier was tasked with determining the correct frequency class for each “on” period, where the possible classes corresponded to the three stimulation frequencies (e.g., 9, 14, and 33 Hz).

**Fig. 7:**
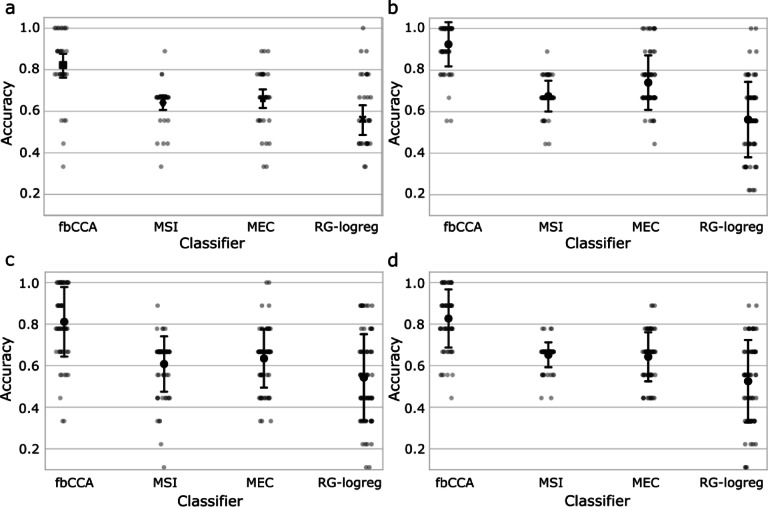
Classification accuracy for each of the 4 textured stimulus conditions (**a**) Worms (**b**) Voronoi (**c**) Static (**d**) Wood Grain for a series of 4 different classifiers. Bars represent ± STD.

**Fig. 8:**
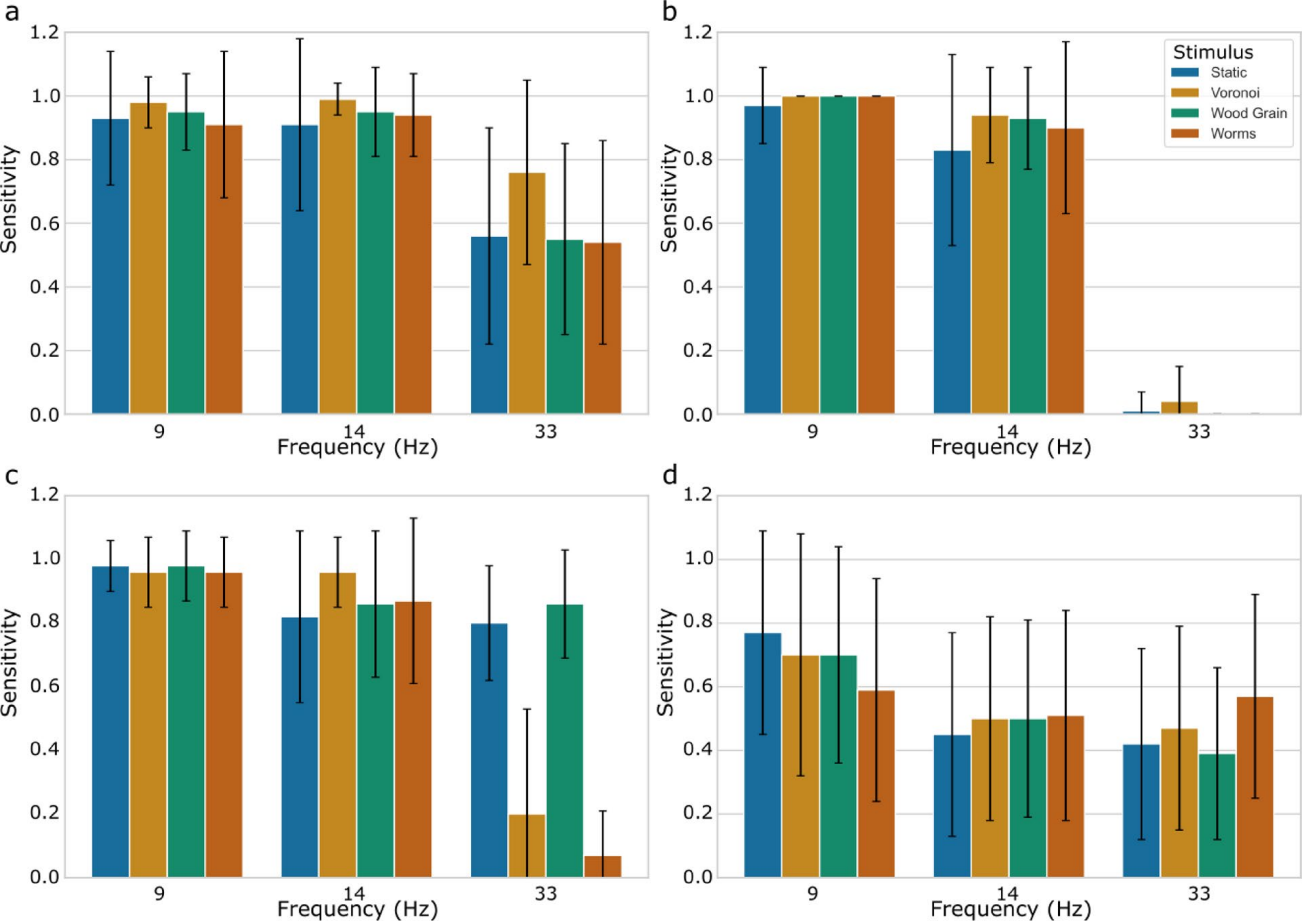
Sensitivity for each of the 4 textured stimulus conditions at each of the 3 frequencies (Hz) for 4 different classifiers: (**a**) fbCCA (**b**) MSI (**c**) MEC (**d**) RG-logreg. Bars represent ± STD.

The fbCCA classifier had the best performance, maintaining a mean classification accuracy greater than 80% for all four textured stimuli. The MSI and MEC classifiers had an intermediate performance, with mean CA values between 60 and 70% for all textured stimuli except for the Voronoi stimulus in the MEC classifier- having a mean CA of 77%. The RG-logreg classifier performed the worst out of the four classifiers, with all textured stimuli having a mean CA below 60%. All values are visualized in Fig. 7. Results of sensitivity analysis are displayed in Fig. 8.

## Discussion

Textured stimuli were investigated as a suitable alternative to traditional high contrast solid color stimuli to alleviate visual fatigue and promote more comfortable use of SSVEP-based BCIs. Our findings indicated that while textures were perceived to be more comfortable than high contrast solid color stimuli, they do not appear to recruit the higher-order visual processing systems as measured by EEG.

Subjective measures of perceived comfort revealed that, on average, the textured stimuli were more comfortable than any of the standard BCI stimuli (maximum/minimum contrast, and checkerboard). This supports our hypothesis that textured stimuli would be perceived as more comfortable than state-of-the-art flashing stimuli and is likely due to the lower contrast content of the textured stimuli, as compared to maximum contrast or checkerboard stimuli.

Interestingly, despite having higher within-image contrasts, the averaged textured stimuli were also perceived as more comfortable than the minimum contrast stimuli. One explanation may relate to higher complexity of the stimulus design, i.e. more “stuff to look at/focus on” in the textured stimuli. Although the comfort scale was intended to measure only the perceived comfort of stimuli, sustained attention and boredom during the experiment may have also played a role in determining final comfort ratings. The textured stimuli may provide a more engaging stimulus than a solid color square.

Looking at the individual textured stimuli, the finding that the Voronoi stimulus was perceived as the least comfortable of the four is unsurprising, as its composition has relatively higher contrast content compared to the other textured stimuli (Fig. 1).

The finding that all stimuli presented at 14 Hz were perceived as less comfortable than stimuli presented at 9 Hz is in line with previous work demonstrating intermediate frequency SSVEP stimuli typically are the most likely to contribute to visual fatigue^2^. It was expected, however, that stimuli presented at 33 Hz would be more comfortable than either of the other frequencies. One likely explanation for this finding is the lack of randomization in the order of frequencies during stimulus presentation. Because the 33 Hz stimuli were presented last during each set, boredom likely set in, influencing comfort scores as a result.

SNR scores were divided into 4 channel subsets with the goal of investigating the effect that textured stimuli may have in eliciting SSVEP signals in the temporal lobe. When investigating averaged textured stimuli against the commonly used solid color and checkerboard stimuli, we expected to see an effect of stimulus type in the T channel subset, but this was not the case. The results indicate that the selected textured stimuli in this work do not recruit processes in the temporal lobe as originally hypothesized, at least not to a sufficient degree to alter system performance. It is possible that the use of the flashing textured image examined in this study does not induce a sufficient change in temporal lobe activation, or that the change in activation does not directly translate to a detectable SNR difference. It was determined, however, that the effects of stimulus type and frequency were significant for the O and OT channel subsets, supporting established knowledge that the SSVEP is elicited in these regions of visual cortex^17^.

In both the O and OT channel subsets, the maximum contrast stimulus generally had higher SNR values than the averaged textured stimuli. Maximum contrast stimuli are known to elicit robust SSVEP signals in the brain at all intermediate frequencies, with this response diminishing as the stimulation frequency increases^2^. The finding that the maximum contrast stimulus had higher SNR values than the textured stimuli at 9 Hz and 14 Hz, but not at 33 Hz in the O channel subset is in line with this. It was observed, however, that the maximum contrast stimuli had SNR values higher than the average of the textured stimuli at 33 Hz in the OT channel subset, suggesting that the maximum contrast stimuli elicited a response in the temporal channels that positively contributed to the total SSVEP signal. An alternate explanation is that textured stimuli elicited such poor signals in the T channels when presented at 33 Hz, that the OT channel SNR values were dragged down.

The result that minimum contrast stimuli SNR values were higher than the averaged textured stimuli SNR values at 9 Hz in the O and OT channel subsets was surprising. The minimum contrast stimuli and textured stimuli have similar contrasts, with the textured stimuli having a slightly higher within-image contrast, especially in the case of the Voronoi stimuli. This finding leads to the idea that contrast content within the stimulus image does not translate into a neural response in the same way that contrast content between the stimulus image and the background does, especially at lower SSVEP frequencies.

The checkerboard stimulus generally had higher SNR values than the averaged textured stimuli. Previous work^36^ supports this finding: performance of a black-background checkerboard stimuli, similar to the one presented in this experiment, was shown to have a high mean information transfer rate when presented at 40 Hz, demonstrating that the performance of a checkerboard stimulus may be well suited for presentation in the high frequency range of SSVEP.

The maximum contrast stimuli having the lowest SNR values of the four stimulus types at 14 Hz in the OT channel subset is an unexpected and somewhat unusual finding. The results shown in Fig. 5 suggest that the temporal channels may have a noisier response to the maximum contrast stimuli, but not the textured, minimum contrast, or checkerboard stimuli at intermediate frequencies.

The discovery that significant effects of stimulus type were only present in the O channel subset when examining the individual textured stimuli further provides evidence against the hypothesis that textured stimuli recruit processing mechanisms in the temporal lobe, at least for static flashing (i.e. non-moving) textured stimuli. This finding, however, does support the idea that textured stimuli may be able to elicit responses in area V2 of the visual cortex. The Voronoi stimuli had higher SNR values than the Worms stimuli at 9 Hz and 33 Hz, but not 14 Hz. The higher contrast content of the Voronoi stimuli likely explains the increased SNR compared to the Worms stimuli, although the absence of this result at 14 Hz or in the Wood Grain and Static stimuli is unexpected and unclear. Based on classification as correlated textured images^13^, the Voronoi, Worms, and Wood Grain stimuli were expected to show an increased SNR value in the temporal lobe, but this was not observed. Future investigations into reliability of SNR outcomes for more variations of the flashing static stimuli (i.e. multiple versions of each textured image) could be investigated to help better elucidate this effect.

We tested a series of classifiers to estimate the impact of different stimuli on BCI classification at various stimulation frequencies and with different classifiers. Within each classifier, the CA was relatively consistent across different textures, with only slight variations, such as the slightly higher accuracy observed for the Voronoi stimulus (Fig. 7). This consistency indicates that the main differences in classification performance arise from the frequency of stimulation and the specific classifier employed, rather than the type of textured stimulus.

Of the classifiers tested, fbCCA performed the best, and MSI, MEC, and RG-logreg had intermediate performance (Fig. 7). Sensitivity analysis revealed that these differences in classification accuracy were largely a result of reduced sensitivity at higher frequencies.

All classifiers demonstrated reduced sensitivity at the highest frequency tested (33 Hz), underscoring a general preference for lower frequencies across the board. The extent of this decline varied among the classifiers. The fbCCA classifier, while achieving classification accuracy above 80% for all textured stimuli (Fig. 7), showed a marked drop in sensitivity at 33 Hz, particularly for the Worms, Static, and Wood Grain textures, where sensitivity fell to 50–60% (Fig. 8). Like fbCCA, the MSI, MEC, and RG-logreg classifiers also had reduced sensitivity with higher frequency stimuli, with significant drops in sensitivity at 33 Hz (Fig. 8). They generally maintained better sensitivity at lower frequencies, though their overall performance was hindered by the limited dataset—only nine 17-second periods per stimulus—suggesting that more data might be needed to improve their efficacy. In the case of the RG-logreg classifier, the small amount of data was particularly impactful as this was the only classifier that was not trainer-free. Generally, SSVEP-based BCIs use epochs of less than 2 s and train on upwards of 10 epochs per class. Our classifier only used 2 epochs of 17 s per class for training, and likely had lower classification accuracy as a result. Because the goal was to identify if textured stimuli are usable with state-of-the-art classifiers, rather than to determine the best possible classifier, the option to split the data into smaller epochs was disregarded.

Overall, the key variation in performance appears to be driven by the interplay between the classifier type and the frequency of stimulation, rather than the specific textured stimulus. While the fbCCA classifier stands out as a strong candidate for SSVEP-based BCI applications, particularly at lower frequencies, the MSI, MEC, and RG-logreg classifiers may benefit from further refinement and additional data to enhance their performance, especially at higher frequencies.

### Limitations

Several limitations must be considered. Firstly, we only displayed one flashing SSVEP stimulus on the computer screen at a time, limiting the applicability of the classifier and SNR results to traditionally described SSVEP-based BCI systems. Although only a single stimulus was presented at a time, this should not affect the implementation of textured stimuli into a multi-stimulus SSVEP BCI system, if the stimuli are placed a minimum distance apart^37^. Additionally, only a limited, and somewhat randomly chosen, set of textured stimuli was evaluated. Textures were chosen to resemble components of natural scenes, but there were no specific exclusion or inclusion criteria to establish what a “textured stimulus” should be composed of or what its composition should look like in the context of this study. The relatively small amount of data recorded needs to be taken into account when considering classifier performance. A balance must be struck between stimulus presentation time, data processing requirements, and participant comfort. Finally, while the order of stimulus types was randomized during the stimulus presentation session, the flashing frequencies were not. Further randomization of presentation order would hopefully remove any bias in reported comfort scores resulting from increased boredom of participants as stimulus presentation progressed.

### Future work

To further evaluate the feasibility of textured stimulus use in SSVEP-based BCI systems, several additional steps must be taken. An offline BCI system was used in this experiment, so practical performance of textured stimuli in real time was not evaluated. Real time performance is key for extending this work to the population of children with severe motor impairments - the ultimate goal of developing comfortable stimuli. Additionally, although we hypothesize that the textured stimuli recruit visual processing mechanisms in area V2 as well as area V1, measurement of brain signals with EEG does not allow for an investigation into the spatial components of the SSVEP signal. To ascertain whether textured stimuli activate V2 more strongly than high contrast solid color stimuli, a neuroimaging method with greater spatial resolution, such as fMRI must be utilized. Finally, in this experiment, textured stimuli were presented as flashing images, with the textured components within each image remaining in the same place. Work investigating the use of dynamically moving textured stimuli may elicit a more robust SSVEP response.

This study has shown the potential of textured stimulus use in SSVEP-based BCI by children over a broad age range (5–18). Although participants with neuropsychiatric disorders, such as attention-deficit hyperactivity disorder were excluded from this experiment, there are characteristics common to different age groups, such as an increase in sustained attention with an increase in age^38^. To investigate possible differences in comfort and SSVEP response due to age, future work should consider subdividing pediatric participants into more specific age groups and measuring more complex confounds such as attention.

## Conclusion

Textured stimuli show promise for use as a more comfortable alternative to state-of-the-art stimuli in SSVEP-based BCI systems. Perceived comfort and high classification accuracies lay the groundwork for their evaluation in an online BCI system and eventual adoption as a more comfortable and adaptable type of SSVEP stimulus. Consideration of comfort is vitally important when developing BCI systems, especially systems intended to be used by children, especially those with complex needs. Current discomfort associated with SSVEP stimuli may be a barrier to the development and use of life-changing BCI technology for children who are unable to interact with their environment. We hope that the development of more comfortable stimuli will be a step toward making SSVEP-based BCI use more fun and engaging.

## Data Availability

Data will be available from the authors (emily.schrag@ucalgary.ca) upon reasonable request.
